# Lifetime Socioeconomic Position and Twins' Health: An Analysis of 308 Pairs of United States Women Twins

**DOI:** 10.1371/journal.pmed.0020162

**Published:** 2005-07-26

**Authors:** Nancy Krieger, Jarvis T Chen, Brent A Coull, Joe V Selby

**Affiliations:** **1** Department of Society, Human Development and Health, Harvard School of Public Health, Boston, Massachusetts, United States of America,; **2** Department of Statistics, Harvard School of Public Health, Boston, Massachusetts, United States of America,; **3** Division of Research, Kaiser Permanente, Oakland, California, United States of America; University of California, Los AngelesUnited States of America

## Abstract

**Background:**

Important controversies exist about the extent to which people's health status as adults is shaped by their living conditions in early life compared to adulthood. These debates have important policy implications, and one obstacle to resolving them is the relative lack of sufficient high-quality data on childhood and adult socioeconomic position and adult health status. We accordingly compared the health status among monozygotic and dizygotic women twin pairs who lived together through childhood (until at least age 14) and subsequently were discordant or concordant on adult socioeconomic position. This comparison permitted us to ascertain the additional impact of adult experiences on adult health in a population matched on early life experiences.

**Methods and Findings:**

Our study employed data from a cross-sectional survey and physical examinations of twins in a population-based twin registry, the Kaiser Permanente Women Twins Study Examination II, conducted in 1989 to 1990 in Oakland, California, United States. The study population was composed of 308 women twin pairs (58% monozygotic, 42% dizygotic); data were obtained on childhood and adult socioeconomic position and on blood pressure, cholesterol, post-load glucose, body mass index, waist-to-hip ratio, physical activity, and self-rated health. Health outcomes among adult women twin pairs who lived together through childhood varied by their subsequent adult occupational class. Cardiovascular factors overall differed more among monozygotic twin pairs that were discordant compared to concordant on occupational class. Moreover, among the monozygotic twins discordant on adult occupational class, the working class twin fared worse and, compared to her professional twin, on average had significantly higher systolic blood pressure (mean matched difference = 4.54 mm Hg; 95% confidence interval [CI], 0.10–8.97), diastolic blood pressure (mean matched difference = 3.80 mm Hg; 95% CI, 0.44–7.17), and low-density lipoprotein cholesterol (mean matched difference = 7.82 mg/dl; 95% CI, 1.07–14.57). By contrast, no such differences were evident for analyses based on educational attainment, which does not capture post-education socioeconomic position.

**Conclusion:**

These results provide novel evidence that lifetime socioeconomic position influences adult health and highlight the utility of studying social plus biological aspects of twinship.

## Abstract 2

Differing socioeconomic positions in adult life are associated with differences in the health of twins raised together.

## Introduction

Twins have long provided a unique opportunity to study how health is shaped from conception to death by biological and social factors [1–5]. At issue are the contributions, singly and combined, of genetic inheritance, in utero postzygotic events before and after twinning, and familial plus societal contexts, including the ways in which twins are treated by family members, each other, and society at large [1–7].

To explore these issues, one major trend in twin research has focused on comparing health status of twins raised separately since birth or early childhood [1–7]. Far fewer studies have investigated how twins raised together, but who differ in their postadolescent socioeconomic position, compare on adult health status [2,4]. Yet such research could potentially inform current debates over the contribution of lifecourse socioeconomic conditions to adult health [8–11], given twins' shared genetic inheritance and early life socioeconomic plus biological exposures. Twins afford an important opportunity to examine the additional impact of adult experiences on adult health in a population matched on early life experiences.

In particular, one important unresolved issue in the burgeoning literature on lifecourse analysis of health concerns how well early life social circumstances are measured, since this these data are essential for distinguishing between the influence of early life and adult conditions on adult health status [8,9]. At issue are the often limited data on childhood socioeconomic conditions [8,9], plus the possibility of systematic error, by adult socioeconomic position, when adults recollect their early childhood circumstances [12–14]. Limited data, recall bias, and poor measurement together hinder obtaining accurate effect estimates, due to confounding by unmeasured factors. This concern is especially salient for studies investigating the social patterning of health, precisely because living and working conditions influence health through myriad discrete yet entangled pathways [8,15].

Further complicating analysis of the impact of childhood and adult socioeconomic position on health are choices regarding the socioeconomic measure(s) employed [16–18]. As discussed in several comprehensive review articles [16–18], considerable evidence exists demonstrating that different socioeconomic measures—e.g., education, occupation, income, wealth, housing tenure, etc.—are not simply “exchangeable” with each other and instead often yield different estimates of the magnitude of the socioeconomic gradient and affect health by independent as well as correlated pathways. For example, while education has often been valued as a socioeconomic measure precisely because, once achieved, it is not subject to reverse causation (e.g., poorer health leading to lower income), it also has been shown to be insensitive to subsequent changes in adult socioeconomic position (e.g., income dynamics) that also can affect adult health status [16–22]. An important implication is that studies concerned with the joint impact of childhood and adult socioeconomic position on health must take into account how their choice of socioeconomic measures may influence their results.

Of note, studies of adult twins, and especially monozygotic twins, can usefully address problems of capturing early life circumstances and assessing the contribution of childhood and adult conditions on adult health. This is because monozygotic twins raised together through childhood (a) are tightly matched on both genetic endowment and the socioeconomic circumstances characterizing their gestation and early life and childhood household resources [4–6], and (b) are the same biological sex, so they are likely to be accorded the same gender expectations and not have differential treatment or access to household resources because of their gender [6,15]. Thus, even without any measurement of childhood conditions, a comparison of adult monozygotic twins who are concordant versus those who are discordant on adult socioeconomic position allows ascertainment of the extent to which socioeconomic position after childhood affects adult health, above and beyond the impact of childhood socioeconomic position. Twin analyses employing diverse socioeconomic measures capturing circumstances earlier versus later in adult life could also potentially yield insight into the impact of cumulative socioeconomic position on health, with interpretation of results for monozygotic twins, including patterns of within-pair variability, aided by comparison to results to same-sex dizygotic twins.

Thus, our objective, framed by ecosocial theory and its concern with the lifelong embodiment of social conditions [15,23], was to compare health status among a cohort of monozygotic and dizygotic women twins with shared upbringing (at least until age 14) and concordant versus discordant adult socioeconomic position. Outcomes included biological markers, anthropometric and health outcomes, and health behaviors. We employed data on both adult occupational class and educational level, hypothesizing that the former might capture relevant aspects of socioeconomic position occurring after completion of educational attainment.

## Methods

### Study Population

The study twin pairs were members of the Kaiser Permanente Women Twins Study Examination II, conducted in 1989–1990 in Oakland, California, United States [24]. The Examination I cohort included 434 twin pairs recruited in 1978–1979 from a twin registry established in 1974 at the Northern California Kaiser Permanente Medical Care Program. All participants resided in the San Francisco Bay Area at the time of Examination I and were born in or prior to 1960 (mean age, 41 y; range, 18–85 y). Zygosity for each pair was determined by analysis of 20 polymorphic loci, such that the probability of misclassification as monozygous was less than 0.001 [24].

For Examination II (1989–1990), original cohort members were sent a self-administered questionnaire on their health and sociodemographic characteristics plus an invitation to return for a physical exam [24]. Cohort retention was high: Only 72 women (8.3%) did not respond, of whom 36 were deceased. Among the 796 respondents, only 87 (10.9%) did not return for a physical exam. After additionally excluding five women whose twin was a nonrespondent, the Examination II cohort included 352 twin pairs (58% monozygotic and 42% dizygotic), representing 81.1% of the original cohort. Enrollment and study of the twins in both cohorts was approved by the Kaiser Permanente Medical Care Program, Northern California Region, Institutional Review board; analyses for this investigation were additionally approved by the Harvard School of Public Health Human Subjects Committee.

### Socioeconomic Data

Childhood and adult socioeconomic position were measured at Examination II using a self-administered questionnaire. We employed a modified version of Erik Olin Wright's occupational class schema [12,16,25–27], analogous to the United Kingdom's newly established National Statistics Socioeconomic Classification system (NS-SEC) [28]. Distinctions, in order of dominance, were between persons classified as “nonworking class” (NWC; own a business and employ others, self-employed, or supervisory employees), “working class” (WC; nonsupervisory employees), or not in the paid labor force [12,16,25–27].

We defined “childhood household social class” as the occupational class position of the person identified as the head-of-household when the respondent was age 14; we also ascertained the proportion of twins who lived together until at least age 14. We measured adult household social class using a validated, gender-appropriate approach, equal to the most dominant class position, taking into account the usual individual class position of the respondent and her partner or other head-of-household, if any [16,25,26]. Using a gender-neutral approach to measuring household class is increasingly recognized as a more valid means of assessing household socioeconomic position than one which automatically assigns it to either (a) the respondent, whether a woman or man, or (b) the occupation of the adult man in the household (if one is present), given the rise of dual wage-earner households [16–18,27–32]. For example, the new UK NS-SEC measure explicitly rejects the prior conventional practice of “males taking precedence over females” when selecting the “household reference person” for assignment of household class, and instead chooses based upon “the person responsible for owning or renting or who is otherwise responsible for the accommodation,” regardless of gender [29]. We also obtained data on the educational level attained by each twin and their father. No data were available, however, on childhood or adult household income, wealth, or debt, or on the educational level of the mother.

Because the twin pairs were matched, by definition, on socioeconomic position in utero through age 14, we categorized twin pairs in relation to adult socioeconomic position. For adult household social class, three types of pairs were possible: two concordant (both WC:WC or NWC:NWC) and one discordant (WC:NWC). For education, the pairs were defined in relation to being concordant or discordant for fewer than 4 y versus 4 y or more of college.

### Health Outcome Data

The selected health outcomes were chosen because of their well-documented associations with socioeconomic position and because risk could plausibly be affected by both early life and adult circumstances [8,9,25]. Self-report data were analyzed for self-rated health (dichotomized as excellent/good versus fair/poor) and medication use. A validated interviewer-administered questionnaire was used to obtain data on physical activity (kcal per kg per y); this instrument assessed the typical amount of time spent in activities of varying intensity at home, at work, and during recreation [33].

Data on anthropometric and biological characteristics were obtained by physical examination and laboratory analysis [24]. Height was recorded to the nearest 0.5 cm, weight was measured to the nearest 0.1 kg, and these data were used to calculate body mass index (BMI, in kg/m^2^). Participants' minimum waist girth was measured using a steel tape at the natural indentation or at a level midway between the iliac crests and the lower edge of the rib cage if no natural indentation was present; hip girth was measured at the level of the greatest protrusion of the buttocks. These measurements were recorded to the nearest 0.5 cm, and the averages of two measures (different by no more than 1 cm) were used to calculate the waist-to-hip ratio (WHR). After participants rested for 5 min, a mercury sphygmomanometer was used to take two measures each of systolic and diastolic blood pressure (seated, right arm); averages of these two measures were used for data analysis. High blood pressure was defined as systolic blood pressure 140 mm Hg or higher, or diastolic blood pressure 90 mm Hg or higher, or taking antihypertensive medication.

Blood for lipid and lipoprotein measurement was obtained after participants had fasted overnight. It was collected into tubes containing ethylenediaminetetraacetic acid (EDTA). Total, high-density lipoprotein (HDL), and low-density lipoprotein (LDL) cholesterol were measured by standard methods [24]. Glucose level (mg/dl [amount × 0.0555 = mmol/l]; measured 2 h post-load) was determined using the glucose oxidase method [24]; analyses using data on glucose levels excluded the five twin pairs for whom one or both twins had values 300 mg/dl or higher.

### Data Analysis

First, to establish the analytic cohort, we identified twin pairs for whom we could determine both that they had lived together until at least age 14 and their joint socioeconomic trajectory (*n* = 308 pairs). This analytic cohort excluded 44 twin pairs (21 missing data on duration lived together; one reporting separation prior to age 14; two where one twin said below age 14 and the other age 14 or above; plus 20 pairs for whom the joint data on adult household class was either missing, inconsistent, or not in the labor force). Second, we ascertained the retained twins' sociodemographic and health characteristics. Third, for continuous outcomes, we calculated (a) the mean matched difference for twin pairs discordant on adult socioeconomic position, setting the twin with the most socioeconomic resources as the baseline, so as to determine both the magnitude and direction of differences in the outcomes among these pairs, and (b) the mean matched absolute difference for twin pairs in each socioeconomic stratum, to ascertain the variability of outcomes among the twin pairs both discordant and concordant on adult socioeconomic position. Additionally, for the categorical outcomes, we calculated the kappa statistic and associated 95% CI [34]. We do not report data on the 18 WC:WC twin pairs, because small numbers rendered the parameter estimates uninterpretable. All analyses were done in SAS [35].

## Results

As shown in Tables 1 and 2, the sociodemographic and health characteristics of the full cohort (*n* = 352 pairs) and the analytic cohort (*n* = 308 pairs) were quite similar, with about 40% having grown up in working class households and 80% in households in which the father had less than a 4-y college education. At Examination II, 32% of the twin pairs in the analytic cohort were discordant for adult household occupational class, and 20% were discordant for individual college attainment.

**Table 1:**
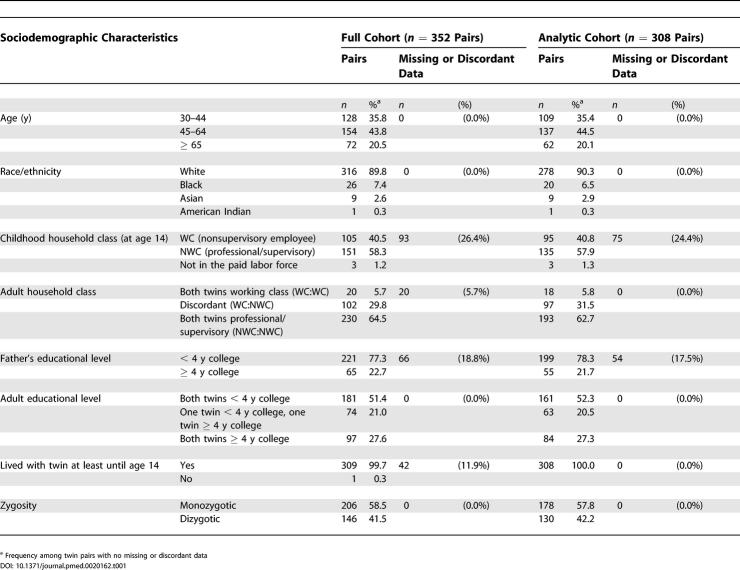
Sociodemographic Characteristics: Kaiser Permanente Women Twins Study, Oakland, California, United States, 1989-1990, Full Cohort (n = 352 Pairs) and Analytic Cohort (n = 308 Pairs)

**Table 2:**
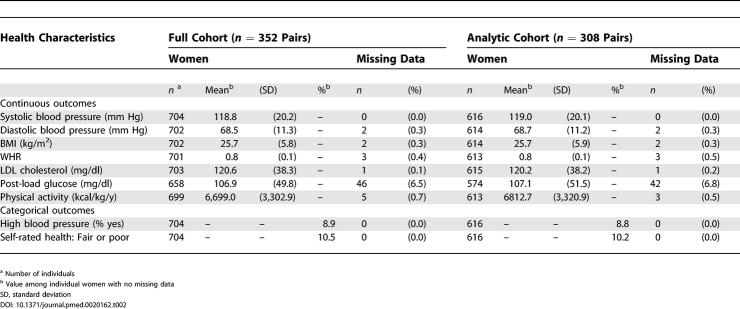
Health Characteristics: Kaiser Permanente Women Twins Study, Oakland, California, United States, 1989-1990, Full Cohort (n = 352 Pairs) and Analytic Cohort (n = 308 Pairs)

Results pertain to both the direction and magnitude of the difference in health status among the twin pairs, in relation to both different measures of adult socioeconomic position and zygosity. First, regarding the magnitude of health disparities among monozygotic twins discordant on adult socioeconomic position, for the analyses using data on adult occupational class (Tables 3 and 4), the WC twin had significantly higher systolic blood pressure (mean matched difference = 4.54 mm Hg; 95% CI, 0.10–8.97), diastolic blood pressure (mean matched difference = 3.80 mm Hg; 95% CI, 0.44–7.17), and LDL cholesterol than her NWC twin (mean matched difference = 7.82 mg/dl [amount × 0.0259 = mmol/l]; 95% CI, 1.07–14.57). Additionally, among the monozygotic twin pairs, a greater proportion of twin pairs discordant on occupational class were discordant for self-reported health compared to twin pairs concordant on occupational class (27.5% versus 6.9%; *p* = 0.0178). Poorer health was also more likely to be reported by the working class twin; among the 51 monozygotic pairs discordant on class, the proportion of pairs in which the WC twin reported fair or poor health while her NWC twin reported excellent or good health (17.6%) was almost twice that of the converse (9.3%, i.e., pairs in which the WC twin reported good or excellent health and the NWC twin reported fair or poor health).

**Table 3:**
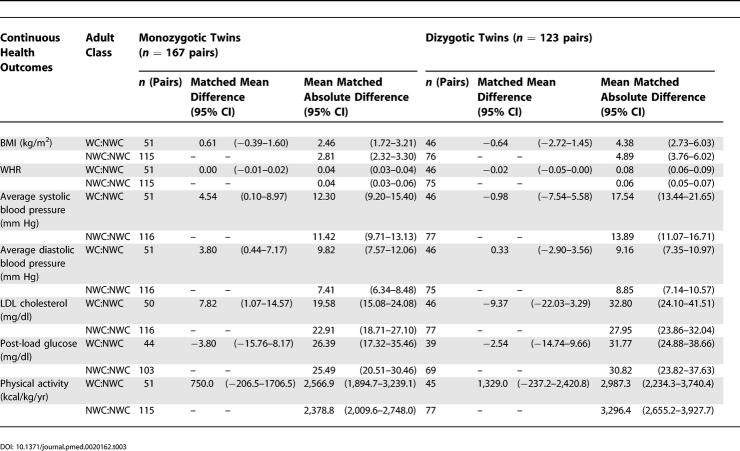
Comparison of Health Outcomes for 290 Twin Pairs Concordant and Discordant on Adult Household Occupational Class (WC and NWC): Continuous Outcomes, by Zygosity: Kaiser Permanente Women Twins Study, Oakland, California, United States, 1989-1990

**Table 4:**
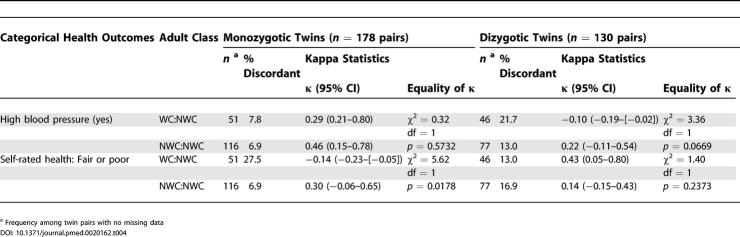
Comparison of Health Outcomes for 290 Twin Pairs Concordant and Discordant on Adult Household Occupational Class (WC and NWC): Categorical Outcomes, by Zygosity: Kaiser Permanente Women Twins Study, Oakland, California, United States, 1989-1990

By contrast, corresponding analyses using data on educational level (Tables 5 and 6) revealed little difference in patterns of health among monozygotic twin pairs discordant on educational attainment. Dizgyotic twins discordant on adult socioeconomic position, whether categorized by occupational class or educational level, likewise did not notably differ on their adult health status (Tables 3–6).

**Table 5:**
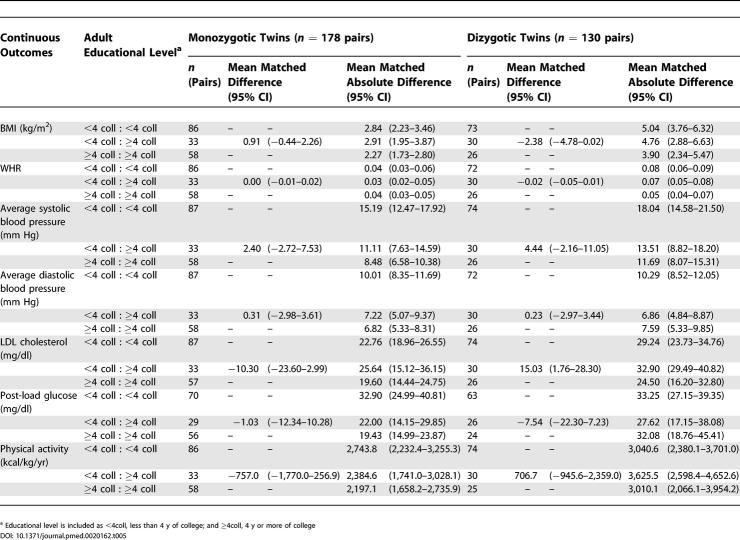
Comparison of Health Outcomes for 290 Twin Pairs Concordant and Discordant on Educational Level (Less Than Versus at Least 4 y of College): Continuous Outcomes by Zygosity: Kaiser Permanente Women Twins Study, Oakland, California, United States, 1989-1990

**Table 6:**
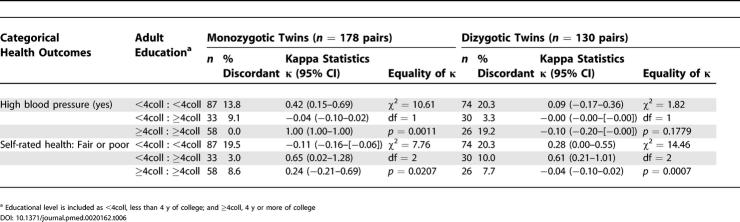
Comparison of Health Outcomes for 290 Twin Pairs Concordant and Discordant on Educational Level (Less Than Versus at Least 4 y of College): Categorical Outcomes by Zygosity: Kaiser Permanente Women Twins Study, Oakland, California, United States, 1989-1990

Second, regarding the variability in health outcomes among twin pairs in relation to their adult socioeconomic position, the mean matched absolute difference was similar among both monozygotic twins who were discordant and concordant on occupational class, and also was similar among dizygotic twins discordant and concordant on occupational class (Tables 3 and 4). Within occupational class strata, however, for all the continuous outcomes other than diastolic blood pressure, the magnitude of variability typically was greater for the dizygotic than the monozygotic twin pairs (Tables 3 and 4). Similar results were obtained for analyses based on educational level (Tables 5 and 6), with some important exceptions. Specifically, for several outcomes among the monozygotic twins, especially average systolic and diastolic blood pressure, post-load glucose, and physical exercise, variability was greatest among twin pairs in which both had fewer than 4 y of college, intermediate among discordant pairs, and least among those where both had 4 y of college or more.

## Discussion

Our study provides novel evidence suggesting that correlations in health outcomes among adult women twin pairs who lived together through childhood vary by their subsequent socioeconomic position, with results sensitive to choice of socioeconomic measure. Although small numbers limit precision of estimates, cardiovascular factors differed more among twins who were discordant on adult occupation class than twin pairs concordant on being professionals, and, within twin pairs discordant on occupational class, the working-class twin typically fared worse than the professional twin. These patterns were much weaker or not evident for analyses using data on educational attainment. Together, these results, combined with our prior research showing that the twins who experienced cumulative deprivation had the worst health [26], lend additional support to the hypothesis that cumulative experiences across the lifecourse, including those after adolescence and after completion of educational attainment, and not just early life experiences, shape adult health [8,9].

Additionally, the greater magnitude of variability in outcomes among dizygotic compared to monozygotic twins within the same socioeconomic strata is what would be expected, given the tighter matching on genetic endowment among the monozygotic twins [3–5]. However, the suggestive finding of greater magnitude of variability, within both the monozygotic and dizygotic twins, among pairs with the least education compared to the most education, especially for the cardiovascular-related results, has not to our knowledge previously been reported. Given that low educational attainment is highly correlated with low socioeconomic resources during childhood [16–18], our results lend tentative support to the hypothesis that increased variability of physiological traits such as blood pressure may be positively associated with greater early-life and cumulative exposure to economic deprivation [36]. A related body of research suggests that chronic exposure to social stressors associated with socioeconomic deprivation may result in repeated activation—and ultimately harmful dysregulation—of physiological systems that respond to stress, thereby increasing risk of elevated blood pressure, insulin resistance, and visceral fat deposition and thus risk of cardiovascular disease, obesity, and diabetes [37–39].

Study limitations include (a) the relatively small number of twin pairs (albeit similar to other twin studies [3,11]); (b) lack of data on detailed occupational class position over time and on age at obtaining a college degree, plus prior or current data on income, poverty, wealth, and debt; (c) lack of data on gestational age, birth weight, birth order, and whether the twins had shared or separate chorions and amniotic sacs [2,7,11,40]; (d) lack of data on differences in the twins' childhood experiences and exposures (e.g., differential treatment accorded to first- versus second-born twins, and to monozygotic versus dizygotic twins [6]); and (e) lack of data on male twins; in addition, the small number of women twins who were concordant on adult working class position limits generalizability (but not internal validity) of results. Most studies assessing the impact of childhood socioeconomic position on health, however, have relied on occupational and sometimes educational data [8,9,26,41–46], reflecting difficulties in obtaining income data across the lifecourse [16–18].

By contrast, strengths of our study include: (a) biological confirmation of zygosity; (b) identical gestational age; (c) identical biological sex, relevant to gender expectations and gendered exposures (more similar for same- versus opposite-sex twins [5,6]); (d) data on age until which the twins lived together; (e) use of a validated and gender-appropriate household occupational class measure, plus data on education; and (f) measurement of anthropometric and physiologic characteristics, not just self-reported health. Moreover, by focusing on postadolescence divergence of socioeconomic position, the study avoided concerns affecting comparisons of twins raised separately versus together, e.g., difficulties in assessing similarities versus differences of the family of origin versus adoptive family [4–6]. A recent analysis of United Kingdom twins' earnings in relation to educational level additionally underscores the utility of using twin analyses to gauge the impact of childhood and adult socioeconomic conditions, at the individual and the household level, on adult economic and health-related outcomes (e.g., smoking) [47].

Overall, results of this study are in accord with other research suggesting that cumulative exposures related to socioeconomic position, not only genetic inheritance and early life experiences, shape adult health [8–11,26,41–46,48]. As with our findings, these studies typically have documented the strongest joint impacts for outcomes pertaining to cardiovascular health [8–11,26,41–46,48]. Unlike prior research, however, the present study newly employed a same-gender twin design, affording comparatively tight matching on life circumstances through early adolescence, with monozygotic twins additionally matched on genetic inheritance, thereby circumventing important concerns raised about likely unmeasured confounders affecting results of prior studies dependent upon adult recall of—and limited data on—childhood socioeconomic position. Even so, generalizability of results to nontwins could be hampered if twins differ systematically from nontwins on factors influencing associations between socioeconomic position and adult health, as perhaps related to maternal and zygotic characteristics relevant to risk of monozygotic or dizygotic twinning or to exposures contingent upon being a twin in utero (e.g., down-regulation of growth) [2,4–7,49–52].

In summary, creative use of social and biological twin data concerning both social and biological aspects of twinship [1–3,6] has the potential to inform current debates about the impact of lifecourse socioeconomic position on health. Suggesting such investigations could have public health import, prior research has estimated that a reduction of 2 mm Hg in the average diastolic blood pressure in the United States—i.e., about half the difference we observed in the comparison of working class to nonworking class monozygotic twins—would translate to a 17% decrease in hypertension, a 6% reduction in coronary heart disease, and a 15% reduction in risk of stroke and transient ischemic attacks [53]. Given the longstanding fascination with twins [1–3,6], if additional and larger twin studies of economically diverse women and men twins confirmed the relevance of cumulative and intergenerational lifetime socioeconomic resources for health, and were also able to include a wider array of socioeconomic measures (e.g., income, wealth, debt, and mother's education) and data on gestational age and birth weight, the evidence would likely have high policy salience, plus importantly enhance understanding of how embodiment of societal conditions shapes population patterns of health, disease, and well-being [15,23,54].

**Patient Summary**

**Background**

Important controversies exist about the extent to which people's health status as adults is shaped by their living conditions in early life compared to adulthood. These debates have important policy implications, with regard to directing resources for improving health: should they be focused on children, on adults, or both? One obstacle to determining the relative influence of early life compared to adulthood on health is a lack of sufficient high-quality data on childhood and adult socioeconomic position and adult health status. Twins research can be used to answer this question, because for twins raised together their social class early in life (here defined as before age 14) will be the same, and study of monozygotic (identical) twins further allows researchers to look at the impact of living conditions on people with the same genetic background.

**What Did the Researchers Do?**

They looked at how much education each twin had and their social class in later life, and they analyzed these in relation to diverse health outcomes (blood pressure, cholesterol, body mass index) in 308 pairs of female twins recruited in California.

**What Did the Researchers Find?**

They found that the monozygotic twins who differed later in life in their social class tended to have differences in health, with the working-class twin having higher blood pressure and higher cholesterol than her professional counterpart. By contrast, differences in education made no difference to these measures of health.

**What Do These Findings Mean?**

It is already believed that social class in children may affect later health; these results suggest that even individuals who had the same social class in childhood may have different health because of adult social class, including their living conditions after completing their educations. The implication is that interventions to eliminate social inequalities in health must take into account adult as well as childhood living conditions.

**Where Can I Get More Information?**

There are many twin sites on the Web. One site with many links, including to registries, is that of the International Society for Twin Studies. http://www.ists.qimr.edu.au/links.html

## Abbreviations

BMI: body mass index
CI: confidence interval
NWC: nonworking class
WC: working class
WHR: waist-to-hip ratio

## References

[pmed-0020162-b01] Hirsch ND (1930). Twins: Heredity and environment.

[pmed-0020162-b02] Gedda L (1961). Twins in history and science. Translated by Marco Milani-Comparetti. Foreword by Robert M. Stecher.

[pmed-0020162-b03] Boomsma D, Busjahn A, Peltonen L (2002). Classical twin studies and beyond. Nat Rev Genet.

[pmed-0020162-b04] Lichtenstein P, Harris JR, Pedersen NL, McClearn GE (1993). Socioeconomic status and physical health, how are they related? An empirical study based on twins reared apart and twins reared together. Soc Sci Med.

[pmed-0020162-b05] Horwitz AV, Videon TM, Schmitz MF, Davis D (2003). Rethinking twins and environments: Possible social sources for assumed genetic influences in twin research. J Health Soc Behav.

[pmed-0020162-b06] Stewart EA (2000). Towards the social analysis of twinship. Br J Sociol.

[pmed-0020162-b07] Machin GA (1996). Some causes of genotypic and phenotypic discordance in monozygotic twin pairs. Am J Med Genet.

[pmed-0020162-b08] Davey Smith G (2003). Health inequalities: Lifecourse approaches.

[pmed-0020162-b09] Kuh D, Ben-Shlomo Y (2004). A lifecourse approach to chronic disease epidemiology: Tracing the origins of ill-health from early to adult life. 2^nd^ ed.

[pmed-0020162-b10] Barker DJP (1998). Mothers, babies, and health in later life. 2^nd^ ed.

[pmed-0020162-b11] Leon DA (2001). The foetal origins of adult disease: Interpreting the evidence from twin studies. Twin Res.

[pmed-0020162-b12] Krieger N, Okamoto A, Selby JV (1998). Adult female twins' recall of childhood social class and father's education: A validation study for public health research. Am J Epidemiol.

[pmed-0020162-b13] Berney LR, Blane DB (1997). Collecting retrospective data: Accuracy of recall after 50 years judged against historical records. Soc Sci Med.

[pmed-0020162-b14] Lin SS, Glaser SL, Stewart SL (2002). Reliability of self-reported reproductive factors and childhood social class indicators in a case-control study in women. Ann Epidemiol.

[pmed-0020162-b15] Krieger N, Davey Smith G (2004). Bodies count and body counts: Social epidemiology and embodying inequality. Epidemiol Rev.

[pmed-0020162-b16] Krieger N, Williams DR, Moss NE (1997). Measuring social class in US public health research: Concepts, methodologies, and guidelines. Annu Rev Public Health.

[pmed-0020162-b17] Lynch J, Kaplan G, Berkman L, Kawachi I (2000). Socioeconomic position. Social epidemiology.

[pmed-0020162-b18] Berkman LF, Macintyre S (1997). The measurement of social class in health studies: Old measures and new formulations. IARC Sci Publ.

[pmed-0020162-b19] Davey Smith G, Hart C, Hole D, MacKinnon P, Gillis C (1998). Education and occupational social class: Which is the more important indicator of mortality risk?. J Epidemiol Community Health.

[pmed-0020162-b20] Backlund E, Sorlie PD, Johnson NJ (1999). A comparison of the relationships of education and income with mortality: The National Longitudinal Mortality Study. Soc Sci Med.

[pmed-0020162-b21] Duncan GJ, Daly MC, McDonough P, Williams DR (2002). Optimal indicators of socioeconomic status for health research. Am J Public Health.

[pmed-0020162-b22] Macintyre S, McKay L, Der G, Hiscock R (2003). Socio-economic position and health: What you observe depends on how you measure it. J Public Health Med.

[pmed-0020162-b23] Krieger N (2001). Theories for social epidemiology in the 21^st^ century: An ecosocial perspective. Int J Epidemiol.

[pmed-0020162-b24] Mayer EJ, Newman B, Austin MA, Zhang D, Quesenberry CP (1996). Genetic and environmental influences on insulin levels and the insulin resistance syndrome: An analysis of women twins. Am J Epidemiol.

[pmed-0020162-b25] Krieger N, Chen JT, Selby JV (1999). Comparing individual-based and household-based measures of social class to assess class inequalities in women's health: A methodological study of 684 US women. J Epidemiol Community Health.

[pmed-0020162-b26] Krieger N, Chen JT, Selby JV (2001). Class inequalities in women's health: Combined impact of childhood and adult social class—A study of 630 US women. Public Health.

[pmed-0020162-b27] Wright EO (1997). Class counts: Comparative studies in class analysis.

[pmed-0020162-b28] Rose D, Pevalin DJ (2003). A researcher's guide to the national statistics socio-economic classification.

[pmed-0020162-b29] National Statistics (UK) (2002). Household level NS-SEC.

[pmed-0020162-b30] Sorensen A (1994). Women, family and class. Annu Rev Sociol.

[pmed-0020162-b31] Krieger N (1991). Women and social class: A methodological study comparing individual, household, and census measures as predictors of black/white differences in reproductive history. J Epidemiol Community Health.

[pmed-0020162-b32] Arber S (1991). Class, paid employment and family roles: Making sense of structural disadvantage, gender, and health status. Soc Sci Med.

[pmed-0020162-b33] Sidney S, Jacobs DR, Haskell WL, Armstrong MA, Dimicco A (1991). Comparison of two methods of assessing physical activity in the Coronary Artery Risk Development in Young Adults (CARDIA) Study. Am J Epidemiol.

[pmed-0020162-b34] Fleiss JL (2003). Statistical methods for rates and proportions. 3rd edition.

[pmed-0020162-b35] SAS Institute. SAS 9.. http://support.sas.com/software/index.htm.

[pmed-0020162-b36] Himmelstein DU, Levins R, Woolhandler S (1990). Beyond our means: Patterns of variability of physiological traits. Int J Health Serv.

[pmed-0020162-b37] McEwen BS (1998). Protective and damaging effects of stress mediators: Allostatis and allostatic load. New Engl J Med.

[pmed-0020162-b38] Sapolsky RM (2004). Why zebras don't get ulcers. 3^rd^ ed.

[pmed-0020162-b39] Brunner E, Berkman L, Kawachi I (2000). Toward a new social biology. Social epidemiology.

[pmed-0020162-b40] Swerdlow AJ, De Stavola B, Maconochie N, Siskind V (1996). A population-based study of cancer risk in twins: Relationships to birth order and sexes of the twin pair. Int J Cancer.

[pmed-0020162-b41] Heslop P, Smith GD, Macleod J, Hart C (2001). The socioeconomic position of employed women, risk factors and mortality. Soc Sci Med.

[pmed-0020162-b42] Pensola TH, Martikainen P (2003). Cumulative social class and mortality from various causes of adult men. J Epidemiol Community Health.

[pmed-0020162-b43] Hart CL, Smith GD, Blane D (1998). Inequalities in mortality by social class measured at 3 stages of the lifecourse. Am J Public Health.

[pmed-0020162-b44] Langenberg C, Hardy R, Kuh D, Brunner E, Wadsworth M (2003). Central and total obesity in middle aged men and women in relation to lifetime socioeconomic status: evidence from a national birth cohort. J Epidemiol Community Health.

[pmed-0020162-b45] Wamala SP, Lynch J, Kaplan GA (2001). Women's exposure to early and later life socioeconomic disadvantage and coronary heart disease risk: The Stockholm Female Coronary Risk Study. Int J Epidemiol.

[pmed-0020162-b46] Brunner E, Shipley MJ, Blane D, Smith GD, Marmot MG (1999). When does cardiovascular risk start? Past and present socioeconomic circumstances and risk factors in adulthood. J Epidemiol Community Health.

[pmed-0020162-b47] Bonjour D, Cherkas LF, Haskel JE, Hawkes DD, Spector TD (2003). Returns to education: Evidence from U.K. twins. Am Econ Rev.

[pmed-0020162-b48] Pollitt RA, Rose KM, Kaufman JS (2005). Evaluating the evidence for models of life course socioeconomic factors and cardiovascular outcomes: A systematic review. BMC Public Health.

[pmed-0020162-b49] Martin JA, Park MM (1999). Trends in twin and triplet births: 1980–97. Natl Vital Stat Rep.

[pmed-0020162-b50] Alexander GR, Kogan M, Martin P, Papiernik E (1998). What are the fetal growth patterns of singletons, twins, and triplets in the United States?. Clin Obstet Gynecol.

[pmed-0020162-b51] Andrew T, Hart DJ, Snieder H, de Lange M, Spector TD (2001). Are twins and singletons comparable? A study of disease-related and lifestyle characteristics in adult women. Twin Res.

[pmed-0020162-b52] Hall JG (2003). Twinning. Lancet.

[pmed-0020162-b53] Cook NR, Cohen J, Hebert PR, Taylor JO, Hennekens CH (1995). Implications of small reductions in diastolic blood pressure for primary prevention. Arch Intern Med.

[pmed-0020162-b54] Krieger N (2004). Embodying inequality: Epidemiological perspectives.

